# From RNA to DNA: How Cargo Identity Reprograms Lipid Nanoparticle Architecture and Function

**DOI:** 10.1002/adhm.202505261

**Published:** 2026-02-02

**Authors:** Erica Quagliarini, Daniela Pozzi, Giulio Caracciolo

**Affiliations:** ^1^ NanoDelivery Lab Department of Molecular Medicine Sapienza University of Rome Rome Italy; ^2^ Center For Nanotechnology Applied to Engineering (CNIS) Sapienza University of Rome Rome Italy

**Keywords:** DNA, gene delivery, lipid nanoparticles, protein corona, RNA

## Abstract

Lipid nanoparticles (LNPs) have become the leading platform for delivering genetic material, gaining global recognition through the success of mRNA‐based COVID‐19 vaccines such as mRNA‐1273 (SpikeVax, Moderna) and BNT162b2 (Comirnaty, BioNTech/Pfizer). Yet, while RNA‐LNPs have reached clinical maturity, their DNA counterparts remain comparatively underexplored, despite holding great promise for gene replacement and genome‐editing therapies. In this review, we turn the spotlight on DNA‐loaded LNPs, examining how their structure, composition, and biological behavior differ from RNA‐LNPs, their natural point of reference, and from earlier lipid‐based systems such as cationic liposome/DNA complexes (lipoplexes). DNA‐LNPs tend to form larger, more heterogeneous, and often multilamellar particles due to the intrinsic stiffness and high charge density of DNA. These distinctive features call for dedicated design strategies, including the use of cationic lipids, pre‐condensation agents, and optimized PEGylation schemes. Moreover, DNA profoundly influences the biomolecular corona that forms in biological fluids, which in turn shapes immune recognition, circulation, and tissue targeting. By highlighting these unique physical and biological challenges, this review underscores the need to move beyond simply adapting RNA‐based formulations. Instead, a cargo‐informed design approach will be key to unlocking the full therapeutic potential of DNA‐LNPs in next‐generation gene delivery.

## Graphical abstract

The evolution of lipid nanoparticles (LNPs) spans from RNA‐LNPs, used in mRNA vaccines, to DNA‐LNPs, ideal for gene therapies. Emerging bionano architectures, decorated with DNA and plasma proteins, pave the way for advanced DNA‐based therapies that are more stable, targeted, and customizable. (Created with BioRender.com)

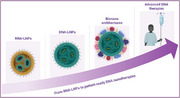

## 1 Introduction

LNPs have emerged as a transformative platform for the intracellular delivery of nucleic acids, gaining widespread attention following their pivotal role in mRNA vaccine development [1]. Their clinical success was demonstrated by the mRNA‐based COVID‐19 vaccines, BNT162b2 (Pfizer‐BioNTech) and mRNA‐1273 (Moderna), which rely on LNPs to encapsulate and deliver mRNA encoding the SARS‐CoV‐2 spike protein [2]. These formulations shared a lipid composition like Onpattro (patisiran) [3, 4], the first FDA‐approved siRNA‐LNP drug for hereditary transthyretin‐mediated amyloidosis, underscoring the versatility and translational potential of this nanocarrier system.

Beyond infectious disease prevention, LNPs are now being investigated as delivery vectors in diverse therapeutic contexts, including cancer, neurological disorders, and rare genetic diseases [5, 6]. Their application spans the delivery of siRNA [7, 8, 9], mRNA [10, 11], and gene editing tools such as CRISPR‐Cas9. The ability to tailor LNP composition to modulate biodistribution via physicochemical tuning has made them an attractive alternative to viral vectors, particularly due to their lower immunogenicity, scalability, and synthetic modularity.

While much attention has been given to LNP design for RNA, the development of DNA‐loaded LNPs (hereafter indicated as DNA‐LNPs) remains disproportionately underdeveloped. On one side, this is surprising given the advantages of DNA as a therapeutic payload: it is chemically more stable than RNA, less sensitive to enzymatic degradation, and enables sustained expression of therapeutic genes following nuclear import [12, 13]. On the other hand, this lack of systematic investigation has left key mechanistic questions unresolved, particularly regarding the causes of low transfection efficiency (TE), poor endosomal escape, and inefficient nuclear entry that continue to limit the effectiveness of LNPs for DNA delivery [14]. One possible explanation for these limitations is that the optimal design principles specific to DNA remain largely undefined. Most formulations are still adapted empirically from RNA platforms, with little consideration for how cargo identity may reshape particle architecture, biological interactions, and functional outcomes. To fully unlock the potential of DNA‐LNPs, it is therefore essential to establish cargo‐specific design principles grounded in a detailed understanding of how DNA influences LNP formation and structure, intracellular behavior, and interactions with the biological environment, which in turn may affect biodistribution, targeting, and immunogenicity [15, 16, 17].

In this review, we focus specifically on LNPs formulated for DNA delivery, without revisiting general principles of RNA‐based systems or features common to LNPs encapsulating either RNA or DNA, which have been thoroughly addressed elsewhere [18, 19]. Our aim is to analyze how the evidence gathered to date has elucidated the physicochemical requirements imposed by DNA on LNP architecture, how these systems behave at the single‐cell level, and what is currently understood about the biomolecular corona that surrounds them. By consolidating emerging insights and outlining key knowledge gaps, we seek to provide a focused perspective that can guide the rational design and optimization of next‐generation DNA‐encapsulating LNPs, moving beyond RNA‐derived paradigms toward truly optimized platforms for non‐viral DNA delivery. Where relevant, comparisons are also drawn with cationic liposome/DNA complexes (lipoplexes), which represent the historical precursors of LNPs and continue to provide valuable mechanistic insight into DNA‐lipid interactions.

**FIGURE 1:**
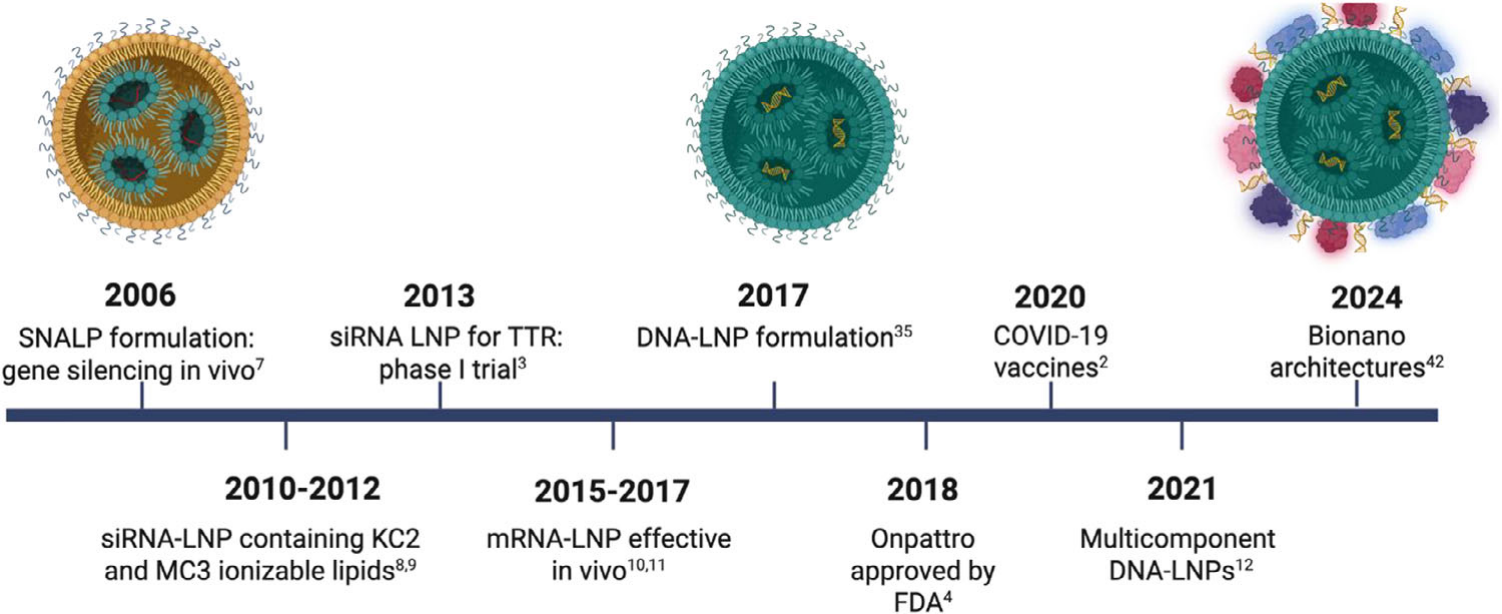
Chronological timeline of key milestones in the development of DNA‐LNPs. (Created with BioRender.com).

The style of this review is not intended to be exhaustively comprehensive. Rather, our aim is to draw on the existing literature to provide a critical perspective informed by the authors’ experience in the field of lipid‐based gene delivery. As a result, not all relevant studies may be cited as would be expected in a traditional systematic review. Instead, the spirit of this work is to highlight selected contributions that, in our view, best capture the scientific progress and conceptual evolution of this research area. In line with this perspective, and to provide historical context for the development of DNA‐LNP technologies, Figure 1 presents a timeline summarizing the key milestones that have shaped the field.

## 2 Physicochemical Properties Shaped by DNA Cargo

LNPs have become a well‐established platform for the delivery of mRNA and siRNA, with a composition and architecture largely optimized around the properties of small, flexible RNA molecules. These standard formulations, such as those used in COVID‐19 vaccines, typically combine ionizable lipids, helper lipids (e.g., DSPC), cholesterol, and PEGylated lipids, yielding LNPs with diameters around 50–100 nm, low polydispersity, and near‐neutral surface charge at physiological pH [20]. Ionizable lipids play a dual role: during synthesis, they acquire a positive charge under acidic conditions to promote efficient electrostatic complexation with the negatively charged nucleic acid cargo, whereas at physiological pH, they become largely neutral, reducing nonspecific interactions and improving tolerability in vivo. PEGylated lipids, on the other hand, contribute to colloidal stability during formulation, control particle size by modulating lipid–lipid interactions, and provide a steric barrier that reduces aggregation and opsonization, thereby extending circulation time after administration. This configuration also favors cytoplasmic delivery and minimizes off‐target interactions. From the very beginning, DNA‐loaded LNPs have been formulated using the same microfluidic protocols originally developed for RNA‐loaded LNPs, as DNA is well known to be chemically stable and to preserve its structural integrity under the mildly acidic conditions employed during LNP assembly [19]. However, when DNA is used as cargo, even within otherwise identical lipid compositions, the resulting LNPs may exhibit markedly different physicochemical properties. These emerging differences underscore the need for a cargo‐specific perspective in LNP formulation. To provide the reader with a clear visual summary of the structural differences that will be discussed in the following sections, we include a side‐by‐side comparison in structure and function between DNA‐LNPs and RNA‐LNPs (Table 1).

**TABLE 1:** Comparative structural and physicochemical features of DNA‐ and RNA‐loaded lipid nanoparticles. The features reported represent general trends emerging from the literature discussed in Section 2. Comparative terms such as higher, lower, larger, or smaller are used throughout the table to indicate relative differences between DNA‐ and RNA‐based formulations. These characteristics should be interpreted as indicative rather than absolute, as deviations may arise depending on lipid composition, formulation strategy, nucleic acid properties, and experimental conditions.

Feature	DNA‐LNP	RNA‐LNP
**Cargo size**	Large plasmids and complex genetic constructs (12, 13, 35).	Small‐to‐medium RNA cargos (1‐11).
**Encapsulation efficiency**	High, typically> 90%. It can be enabled by specific encapsulation strategies such as DNA pre‐condensation with cationic agents (e.g., protamine) (24, 39)	High, typically> 90% (7, 8)
**Cargo stability**	DNA is chemically more stable and resistant to degradation (12, 13).	RNA is less stable and more prone to chemical and enzymatic degradation, requiring stringent storage conditions (5, 23, 30).
**Size and polydispersity**	Larger and more heterogeneous particle populations, often exceeding 150 nm in diameter with PDI higher than 0,2 (12, 13, 35).	Smaller and more homogeneous nanoparticles with size typically around 50 nm with PDI lower than 0,1 (1‐11).
**Zeta‐potential**	Lower N/P ratios may be sufficient to encapsulate DNA (34).	Higher N/P ratios are needed to encapsulate RNA compared with DNA (8).
**Nanostructure**	Multilamellar or toroidal internal organizations that confer increased structural stability at the cost of lower cargo release (12, 13, 42).	Predominantly amorphous or inverted hexagonal phases that facilitate cargo release (45‐47, 49).
**Endosomal escape**	Limited by strong DNA‐lipid interactions and compact internal organization (13, 83).	Enabled by less ordered nanostructure (45‐47, 49, 78).
**Subcellular localization**	Acts in the nucleus, thereby imposing a major barrier to delivery efficiency (13, 50, 61).	Acts in the cytosol with no requirement for nuclear entry, eliminating this barrier but precluding permanent genetic modification (1‐11).
**Expression duration**	Potential for long‐term gene expression, but limited in non‐dividing cells due to difficulty in nuclear import (35).	Rapid and transient protein expression is well‐suited for vaccines and short‐term therapies, but inherently unsuitable for long‐term expression (33).
**Immunogenicity**	Activation of innate immune pathways (e.g., cGAS–STING), which can be advantageous for vaccine applications but undesirable in chronic therapeutic settings (82).	Generally associated with lower innate immune activation (6).

### 2.1 Size and Polydispersity

One of the most immediate and consistently observed effects of DNA encapsulation into LNPs is a substantial increase in particle size. While LNPs formulated for mRNA or siRNA typically fall below 100 nm in diameter, DNA‐loaded LNPs, particularly those encapsulating plasmids or long linear DNA, commonly exceed 150 nm, with several formulations reaching up to 200–250 nm [21]. This behavior can be ascribed to the intrinsic physical properties of DNA, such as its high molecular weight, structural rigidity, and extended conformation in solution, which impose greater spatial constraints during LNP self‐assembly.

Among these features, DNA length appears to play critical roles, as highlighted by recent investigations. In a study conducted by Kazuya et al., extracellular vesicle‐mimicking LNPs exhibited mean diameters in the range of ∼170–190 nm when loaded with plasmid DNA, with size and polydispersity index (PDI) highly dependent on the DNA‐to‐lipid ratio and the mode of encapsulation [22].

A further perspective on the role of DNA length comes from the work of Fan et al., who investigated LNPs encapsulating antisense oligonucleotides (ASOs). While ASOs are considerably shorter than plasmid DNA, their study similarly revealed that suboptimal control of formulation parameters such as mixing ratio, flow rate, and lipid composition could still produce particles with diameters exceeding 200 nm and PDI values above 0.3. This mirrors the trends observed for plasmid DNA‐loaded systems, underscoring that nucleic acid size alone does not prevent the emergence of large, heterogeneous particles when formulation conditions are not well tuned. Only through careful adjustment of ethanol‐to‐buffer ratios and fine control of the microfluidic mixing regime, Fan et al. could produce small‐size (∼145 nm) and uniform (PDI < 0.2) LNPs [23].

The concentration of DNA during formulation also plays a critical role in determining the final particle size of LNPs. Ottonelli et al. demonstrated that mixing pDNA with the aqueous phase prior to LNP assembly enabled the production of monodisperse LNPs (∼150–160 nm, PDI < 0.1) even at relatively high DNA concentrations (up to 15 µg/mL). When the DNA loading was raised to 100 µg/mL, the average particle diameter increased to approximately 250 nm, while polydispersity remained within an acceptable range (PDI < 0.3), indicating that higher DNA content primarily affected particle size without substantially compromising formulation uniformity. By contrast, when DNA was added to pre‐formed empty LNPs (post‐loading), the resulting systems showed sharp increases in both particle size (∼250 nm) and polydispersity (PDI > 0.4), accompanied by significant colloidal instability. This highlights the critical role of DNA incorporation timing and supports the superiority of in situ encapsulation strategies for maintaining particle uniformity and functionality [24]. Mashal et al. independently confirmed these observations in their study of lipid‐peptide‐DNA NP, where non‐laminar or turbulent methods (e.g., the ouzo emulsification method) produced markedly larger and more polydisperse particles with respect to microfluidics synthesis due to the lack of controlled lipid rearrangement and electrostatic stabilization during assembly [25].

Several studies have demonstrated that LNP size and polydispersity are also critically governed by the ratio between the aqueous and organic phases during formulation [12, 26]. For a fixed DNA mass, increasing the volume of the acidic aqueous phase reduces the local DNA concentration at the mixing interface, thereby favouring more controlled nucleation and the formation of smaller, more monodisperse particles. In contrast, decreasing the aqueous volume, or equivalently increasing the DNA concentration while maintaining a constant flow rate ratio (FRR), raises the local charge density experienced by ionizable lipids, promoting rapid nucleation and the emergence of larger, more heterogeneous structures. These effects are further amplified by the intrinsic physical properties of DNA: its large contour length and limited conformational flexibility impose geometric constraints during self‐assembly, favouring less compact packing modes and, consequently, larger particle diameters and broader size distribution. Accordingly, formulation studies involving DNA and other high charge density cargos consistently show that mixing conditions and phase ratios are dominant determinants of particle size, underscoring the importance of treating volume ratios as a key design parameter in DNA‐LNP formulation.

In addition to the effects of DNA itself, LNP particle size is also strongly influenced by the specific lipid composition of the formulation. Notably, the choice between ionizable and permanently cationic lipids is a first determinant of LNP architecture, profoundly influencing particle formation and compactness. Ionizable lipids were originally developed and extensively optimized for the delivery of small and flexible RNA cargos. This design paradigm has been highly successful for RNA‐LNPs, yielding small, homogeneous particles with favorable safety profiles. However, when applied to plasmid DNA, the same ionizable lipid architectures may be less effective in promoting tight condensation and compact packing. The large size, high charge density, and reduced conformational flexibility of DNA impose more stringent requirements on electrostatic stabilization during self‐assembly, often exceeding the condensation capability of ionizable lipids alone. In this context, permanently cationic lipids, such as DOTAP, provide a higher and persistent charge density that favors stronger DNA compaction and more efficient nucleation, resulting in smaller and more uniform particles [27]. Recent evidence from Lim et al. shows that formulations incorporating the permanently cationic lipid DOTAP yielded markedly smaller particles (∼130 nm) than those prepared with ionizable lipids such as DLin‐MC3‐DMA or DODAP, even when formulation conditions were kept identical [28]. This observation, consistent across multiple studies, suggests that cationic lipids may offer enhanced DNA condensation and particle compactness, making them particularly advantageous for efficient and uniform DNA‐loaded LNP design.

Incorporating PEG‐lipids into LNP formulations is a key step for controlling particle size and reducing nonspecific interactions in vivo. Foundational work in the RNA‐LNP field demonstrated that PEG‐lipids act as kinetic stabilizers during rapid self‐assembly, preventing uncontrolled fusion of nascent vesicles and thereby enabling the formation of well‐defined, monodisperse nanoparticles [29]. The steric barrier generated by PEG chains limits lateral lipid‐lipid interactions and modulates curvature during nucleation, ultimately dictating the final particle size and internal architecture. Early studies further showed that even small amounts of PEG‐lipid (1.5 mol%) dramatically narrow size distributions and improve colloidal stability, establishing PEG as an essential excipient for reproducible RNA‐LNP manufacturing [30].

This principle holds for DNA‐loaded LNPs, but its implementation appears more complex. Multiple studies have shown that PEG‐dependent size control is influenced not only by PEG molar fraction but also by the chemical structure of the PEG‐lipid, including the hydrophobic anchor, acyl chain length, and headgroup charge [31]. These structural parameters modulate how PEG‐lipids partition within the outer leaflet during DNA‐induced reorganization, a phenomenon far less pronounced in RNA‐LNPs. Charged PEG‐lipids, particularly anionic PEG‐phosphoglycerides, were significantly more effective in reducing particle size (to ∼50–100 nm), likely by enhancing electrostatic counterbalancing during DNA condensation. In contrast, neutral PEG‐lipids exerted minimal influence on size, suggesting limited contribution to lipid reorganization around the bulky DNA scaffold.

An additional complication unique to DNA‐LNPs is the tendency of high PEG content to increase polydispersity, likely due to PEG‐induced micelle formation or local lipid phase separation at the interface, behaviors previously reported in PEG‐rich siRNA‐LNPs and classical liposomal systems. These effects imply that PEGylation may interfere with DNA‐driven lipid packing to a greater extent than with RNA cargos, whose smaller size and conformational flexibility impose fewer geometric constraints during assembly [32]. Thus, although PEG‐lipids remain indispensable for stabilizing DNA‐LNPs and limiting their size, their influence is more variable and mechanistically intertwined with DNA condensation, lipid redistribution, and cargo‐induced phase behavior. These cargo‐specific sensitivities underscore the need for systematic optimization of PEG identity, PEG density, and lipid anchor chemistry in DNA‐LNP formulations, an aspect that remains less established compared to the mature RNA‐LNP literature.

Collectively, the available evidence demonstrates that incorporation of plasmid DNA typically increases both the hydrodynamic size and the PDI of LNPs. Simply repurposing ethanol injection or microfluidic protocols designed for mRNA frequently leads to broader size distributions, less efficient encapsulation, and poor colloidal stability. Minimizing these effects is not trivial and requires a rigorous optimization process that systematically explores several degrees of freedom, including DNA and lipid concentration, lipid composition, lipid‐to‐DNA molar ratio, microfluidic mixing parameters such as FRR and total flow rate (TFR), and the physicochemical characteristics of the nucleic acid cargo itself (e.g., plasmid size, topology, and sequence‐dependent rigidity). The complex and often non‐linear interdependencies among these parameters preclude reliable a priori prediction of formulation outcomes. The current lack of standardized frameworks for DNA‐LNP design underscores the need for more systematic strategies, such as design‐of‐experiments (DOE) approaches, to accelerate the identification of formulations with robust and clinically translatable physicochemical properties [33].

### 2.2 Zeta Potential

The surface charge of LNPs is another parameter strongly influenced by the presence of DNA. While the intrinsic charge density per nucleotide is similar for DNA and RNA, the molar ratio between positively charged groups (N) and negatively charged phosphate groups (P) (hereafter termed N/P ratio) required to neutralize the genetic cargo and to confer a net positive surface charge to LNPs are not universal but depend strongly on the nature and topology of the nucleic acid being delivered. DNA is most often plasmidic and therefore circular and supercoiled, whereas RNA cargos are typically linear. Early and systematic studies by Junquera and co‐workers demonstrated that supercoiled plasmid DNA exposes a reduced fraction of sterically accessible phosphate groups compared with linear nucleic acids [34]. Therefore, efficient condensation and stable formulation of plasmid DNA can be achieved with a reduced effective requirement for charge compensation with respect to linear RNA molecules. It is therefore reasonable to expect that lower N/P ratios may be sufficient to fully encapsulate DNA compared with RNA in LNP‐based formulations. Consistent with this view, Cullis et al. reported that a typical N/P ratio of approximately 6 is required to produce stable RNA‐LNPs using microfluidic mixing approaches [8]. In contrast, in efforts aimed at optimizing DNA encapsulation, Quagliarini et al. demonstrated that efficient loading and stable DNA‐LNP formulations could be achieved at significantly lower N/P ratios (≈3). Together, these observations provide experimental support for the general principle that nucleic acid topology, i.e., supercoiled versus linear, can play a key role in defining the effective charge compensation required for nanoparticle assembly. Notably, these values should be interpreted as indicative rather than absolute. N/P ratios can be increased beyond the minimum required for encapsulation, leading to alternative physical‐chemical properties. In line with this, Kulkarni et al. demonstrated that increasing the N/P ratio in DLin‐KC2‐DMA‐based LNPs loaded with plasmid DNA reduced particle size, from 130 nm at N/p = 3 to 75 nm at N/p = 6, resulting in particles that shift from mildly negative to near‐neutral or even positive zeta potential as the N/P ratio increases [35]. Consistent with these considerations, a wide range of N/P ratios spanning from 3 to up to 75 has been reported in the literature for DNA‐loaded LNP formulations, highlighting the absence of a single optimal value and the strong dependence of electrostatic balance on formulation conditions [36].

It is interesting to note that the principles outlined above for LNPs are fully consistent with earlier observations reported for lipoplexes, suggesting that mechanistic insights gained from lipoplex‐based systems can be reasonably leveraged to inform the rational design of LNP formulations [37]. Among strategies previously established for lipoplexes, DNA condensation within LNP formulations can be promoted through the use of cationic condensing agents, including arginine‐rich proteins such as protamine [38], naturally occurring polyamines like spermidine (3^+^) and spermine (4^+^), as well as multivalent metal ions (e.g., Mg^2^^+^, Ca^2^^+^, Mn^2^^+^) or trivalent species such as Fe^3^^+^ and Al^3^^+^. Under microfluidic mixing conditions, these positively charged species may enhance electrostatic stabilization, promote tighter packing of the large, rigid DNA molecules, and improve encapsulation efficiency. A recent study demonstrated the benefits of pre‐condensing plasmid DNA with P‐reagent, a commercially available cationic condensing agent, before the microfluidic assembly of LNPs [39]. The rapid mixing within the microfluidic channels promoted the spontaneous self‐assembly of LNPs encapsulating the pre‐condensed DNA, resulting in improved encapsulation efficiency, more compact particle structures, and narrower size distributions compared to formulations prepared without the pre‐condensation step. These modifications also resulted in higher TE. Mechanistic studies indicated that DNA pre‐condensation reduces lysosomal sequestration and favors the perinuclear and nuclear localization of LNPs.

The requirement for stronger DNA condensation has led several research groups to investigate the replacement of ionizable lipids with permanently cationic lipids (such as DOTAP or DDAB) to improve encapsulation efficiency and particle compactness. However, permanently cationic lipids are well known to introduce increased systemic toxicity, driven by persistent positive surface charge, nonspecific interactions with serum proteins, and membrane destabilization in non‐target tissues [40]. To mitigate these risks, their use must therefore be coupled with complementary design strategies aimed at restoring a stealth‐like behavior. These include limiting the molar fraction of permanently cationic lipids, combining them with degradable or pH‐sensitive lipid components, and exploiting cleavable PEG‐lipids that detach after systemic circulation to restore intracellular activity while minimizing off‐target toxicity [41]. A recent study demonstrated that DNA can be added post‐synthesis to the particle surface, acting as an effective surface‐passivating element that mimics the stealth behavior of conventional ionizable lipids [42]. The rationale behind this approach is to invert the surface charge of cationic LNPs as previously done for lipoplexes [43], thereby conferring stealth‐like properties and reducing the undesired adsorption of immunogenic plasma proteins such as immunoglobulins and complement factors.

Collectively, these findings highlight that DNA cargo profoundly reshapes the electrostatic and structural landscape of LNPs, making charge compensation, condensation strategies, and surface engineering critical parameters for their rational design [44, 45]. Yet, these aspects remain only marginally explored and should become the focus of fundamental research efforts aimed at translating the resulting knowledge into the development of optimized DNA‐based nanoparticles suitable for clinical application.

### 2.3 Nanostructure

Foundational work by Pieter Cullis and colleagues established the basis for understanding the nanostructure of siRNA‐loaded LNPs [46]. These siRNA‐LNPs were shown to organize into structured core–shell particles, with the siRNA‐ionizable lipid complex forming the core and helper lipids (e.g., DSPC), PEG‐lipids, and cholesterol populating the outer. Other RNA‐loaded LNPs have been shown to form amorphous [46] or inverted hexagonal (H_II_) phases [47].

The structural and functional characterization of DNA‐loaded LNPs is a more recent field of investigation and, as such, remains less mature and supported by fewer experimental studies compared to RNA‐loaded systems. This relative scarcity of data underscores the need for careful analysis of what is currently known, what remains unclear, and where future efforts should be directed. In this section, we examine the most consolidated findings to date, highlight the critical knowledge gaps, and discuss emerging perspectives that may guide the rational design of optimized DNA‐LNPs.

Theoretical treatments developed by Avinoam Ben Shaul and colleagues clarified that the internal organization of lipid‐DNA complexes reflects a balance between electrostatic interactions, lipid curvature elasticity, and the conformational entropy of the nucleic acid [48]. According to these general principles, architectures of DNA‐LNPs that minimize bending and topological frustration of the nucleic acids are thermodynamically favored. Extended DNA molecules preferentially stabilize lamellar arrangements with low curvature, whereas shorter and more flexible oligonucleotides can be accommodated within highly curved core‐shell or inverted hexagonal phases without incurring a prohibitive elastic penalty.

Advanced characterization techniques, such as cryogenic transmission electron microscopy (cryo‐TEM), synchrotron small‐angle X‐ray scattering (SAXS), and small‐angle neutron scattering (SANS), confirmed that the DNA length has a major effect on the nanoscale organization of LNPs.

Small constructs, such as minicircle DNA or linearized gene fragments, can be compacted within the particle core, leading to tight packing as observed for siRNA‐loaded LNPs. On the other hand, longer DNA, such as calf thymus and supercoiled plasmids, present substantial spatial and topological constraints that can significantly alter the overall architecture of the LNP [42]. Gilbert et al. used SAXS, SANS, and cryo‐TEM to demonstrate that LNPs loaded with calf thymus DNA form ordered, multilamellar domains with characteristic repeat distances (∼5.8 nm), larger than those observed for two model mRNAs, namely polyadenylic acid (polyA) and polyuridylic acid (polyU) [49]. Using synchrotron SAXS, Digiacomo et al. [50] showed that plasmid DNA‐LNPs exhibit periodic lamellar domains (d‐spacing ∼6.8 nm), consistent with lipid bilayers intercalated by hydrated DNA strands.

An unresolved question is whether specific lipid components, such as ionizable lipids, helper phospholipids, cholesterol, and PEG‐lipids, differentially modulate the structural stability of DNA‐LNPs. While this has been extensively studied in lipoplexes, where the adoption of lamellar domains is mainly dictated by the molecular shape of the constituent lipids [51, 52, 53], and partially addressed for siRNA‐LNPs, it remains largely unexplored for DNA cargos.

Among the different lipid species, PEGylated lipids are those whose role in shaping the nanostructure of LNPs has been most extensively investigated. PEGylated lipids are currently an essential component of LNPs as they ensure small particle size and homogeneous distribution during synthesis. However, their use is not without limitations: PEG can trigger immune responses, including the generation of anti‐PEG antibodies, which has raised concerns about its long‐term safety and efficacy [54]. As a result, a growing body of literature is devoted to identifying strategies to minimize PEG content in LNPs or replace it with alternative stabilizing agents. This makes it particularly important to investigate the structural organization of PEG‐free DNA‐LNPs, while also clarifying the specific impact of PEG when small amounts must still be retained, since in many cases complete elimination is not feasible, and the practical objective is reduction rather than full substitution. To this end, several studies have focused on dissecting the role of PEG in modulating the internal structure, stability, and performance of LNPs.

Generally, PEGylated formulations display a less ordered internal arrangement than their unPEGylated counterparts, indicating that PEG chains can perturb the regular lamellar packing. This nanoscale disorganization in PEGylated LNPs, a detail that may escape less experienced eyes but carries significant mechanistic implications, is reminiscent of observations reported by Cyrus Safinya's group in the early 2000 s for lipoplexes [55]. Safinya and coworkers demonstrated that osmotic stress generated by PEG chains promoted the separation of DNA‐rich and PEG‐rich domains, with the DNA‐rich regions exhibiting more densely packed helices.

Despite this structural analogy, some differences between DNA‐LNPs and lipoplexes deserve attention. In SAXS patterns of lipoplexes, a characteristic Bragg peak associated with DNA‐DNA in‐plane correlation is typically observed, serving as a hallmark of DNA packing. By contrast, SAXS profiles of DNA‐loaded LNPs lack this distinctive peak, indicating that DNA does not organize into extended periodic lattices but is instead confined in a more heterogeneous or disordered arrangement. Since shifts in the DNA peak serve as a powerful indicator of structural rearrangements, the lack of an equivalent, well‐defined structural marker makes it difficult to track how LNP nanostructures evolve during intracellular trafficking and interact with cellular membranes. This limitation, in turn, hampers the interpretation of their structural dynamics and the evaluation of how nanoscale organization affects endosomal escape, disassembly, and nuclear delivery.

In sum, the markedly larger hydrodynamic sizes, broader polydispersity, altered surface charge requirements, and the emergence of multilamellar or toroidal nanostructures are not minor formulation deviations but cargo‐imposed structural constraints that fundamentally reshape nanoparticle behavior. These observations strengthen the central message of this review: DNA is not a passive payload but an active structural determinant that shapes LNP morphology, stability, and function. To advance the field, it will be crucial to establish correlations between nanoscale organization, intracellular trafficking, and therapeutic outcomes through the integration of high‐resolution imaging and mechanistic biological assays. The current state of knowledge on this topic, along with recent advances in correlating nanostructure with intracellular trafficking and functional efficacy, will be examined in detail in the following Section 3.

## 3 Cellular Interactions of DNA‐Loaded LNPs

**FIGURE 2:**
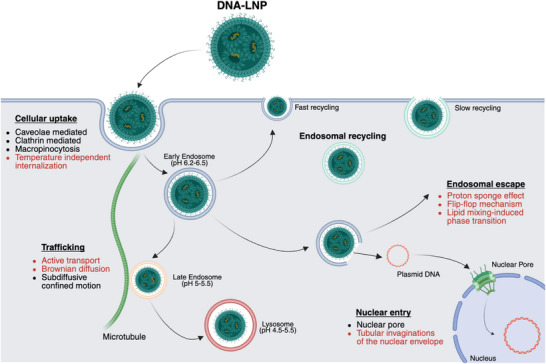
Cellular interactions of DNA‐LNPs. Schematic representation of the cellular uptake and intracellular trafficking pathways of DNA‐loaded lipid nanoparticles (DNA‐LNPs). Following interaction with the plasma membrane, DNA‐LNPs can be internalized through multiple endocytic routes, including caveolae‐mediated endocytosis, clathrin‐mediated endocytosis, macropinocytosis, and temperature‐independent internalization mechanisms. Once internalized, vesicles traffic through early and late endosomal compartments, with possible sorting toward fast or slow recycling pathways, or progression toward lysosomal degradation. Intracellular transport may involve both active, microtubule‐assisted motion and diffusive or subdiffusive dynamics [50, 61]. A critical step for functional delivery is endosomal escape, enabling the release of plasmid DNA into the cytosol, followed by nuclear entry through nuclear pore complexes or invaginations of the nuclear envelope. Mechanisms highlighted in red indicate processes that have been extensively demonstrated for lipoplexes, such as proton sponge‐mediated escape [67], lipid mixing–induced phase transitions [69], and specific active transport modes [56], but that have not yet been conclusively established for DNA‐LNPs. For DNA‐LNPs, these steps remain the subject of ongoing investigation, and their relative contribution to intracellular delivery efficiency is still being elucidated. (Created with BioRender.com).

Cellular interactions of DNA‐loaded LNPs include cellular uptake, intracellular trafficking, endosomal escape, and the nuclear entry of DNA to enable transcription (Figure 2). However, as highlighted by recent evidence [56], intracellular trafficking, endosomal escape, and lysosomal degradation should not be viewed as distinct steps but rather as highly interdependent processes forming a single barrier to efficient gene delivery. This continuum means that the destination of the cargo is inherently linked to the mode of entry and the intracellular route followed, making efficient coordination of uptake, trafficking, and nuclear access particularly critical for DNA‐based systems. For the sake of clarity and for the benefit of the reader, in this section, the available evidence will be discussed sequentially, without explicitly emphasizing the interdependence of these processes.

### 3.1 Cellular Uptake Mechanisms

Early studies on lipoplexes that represent, as above clarified, the precursors of today's DNA‐LNPs, provided key foundational insights, showing that cell internalization primarily occurs via endocytic pathways, including clathrin‐mediated endocytosis, caveolae‐mediated endocytosis, and macropinocytosis [57, 58]. Temperature‐independent fusion with the plasma membrane via lipid reorganization may also contribute to delivery efficiency, particularly in systems formulated with highly fusogenic multicomponent lipid mixtures [59, 60]. These findings were shown to depend on a combination of factors such as lipid composition, molecular shape of the lipids, the number of lipid species included in the formulation, particle size, surface charge, and supramolecular nanostructure. While the contribution of each factor has been extensively investigated, no single, coherent framework has emerged that consistently links all these variables to uptake behavior. Nevertheless, this body of knowledge has been instrumental in identifying formulation strategies that allow a degree of control over cellular internalization.

A similar level of systematic dissection has so far been attempted only in a limited number of studies on DNA‐LNPs that prevalently addressed the role of lipid composition on the cellular internalization mechanism. An illustrative case is provided by work employing image‐based mean square displacement (iMSD) analysis, which demonstrated that PEGylated lipids redirect uptake pathways by favouring caveolae‐ and clathrin‐mediated endocytosis over micropinocytosis [61]. These findings highlight the critical role of PEG in modulating endocytic entry routes and illustrate how subtle modifications to LNP composition can substantially alter intracellular trafficking.

The use of ionizable lipids can affect the mechanisms governing cellular uptake of DNA‐LNPs, depending on whether uptake is evaluated in vitro/ex vivo or in vivo. Under physiological pH conditions, ionizable lipid‐based DNA‐LNPs typically display a near‐neutral net surface charge, which markedly reduces the electrostatically driven internalization mechanisms that are characteristic of permanently cationic nanocarriers. As a result, in vitro or ex vivo cellular uptake is less dominated by nonspecific charge‐mediated interactions with the plasma membrane. In vivo, the role of ionizable lipids becomes even more consequential. By remaining largely neutral in circulation, ionizable lipid‐containing DNA‐LNPs are expected to limit nonspecific adsorption of plasma proteins and, consequently, to recruit a protein corona that differs from that formed on DNA‐LNPs based on permanently cationic lipids. Although systematic comparative studies are still lacking, it is reasonable to assume that these differences in corona composition modulate the interaction of DNA‐LNPs with cell‐surface receptors on target cells, thereby influencing uptake pathways and downstream intracellular processing.

### 3.2 Intracellular Trafficking

A second layer of complexity is introduced by intracellular trafficking dynamics, which have been extensively investigated for lipoplexes using a variety of experimental approaches, most notably single‐particle tracking [62], spatio‐temporal image correlation spectroscopy (STICS) [63], and iMSD analysis [64]. These studies have shown that lipoplexes relying on active transport along microtubules tend to accumulate in lysosomal compartments, where they are exposed to hydrolytic enzymes and undergo degradation, ultimately reducing nucleic acid delivery efficiency [38]. By contrast, particles displaying more stochastic, diffusion‐dominated motion within the cytoplasm are less likely to be sequestered in degradative pathways and may exhibit a higher probability of reaching the nucleus or remaining available for cytosolic translation. Moreover, the specific intracellular route, such as endosomal recycling, retrograde transport to the Golgi, or direct fusion with early endosomes, can markedly shift the balance between degradation and productive delivery.

In the case of LNPs, this aspect has so far been explored only to a limited extent. To date, iMSD‐based analyses have been applied to DNA‐loaded LNPs in a small number of studies, primarily to dissect how specific formulation parameters modulate intracellular motion and fate. For instance, Digiacomo et al. employed iMSD to elucidate the impact of PEGylation on the intracellular dynamics and confinement of DNA‐LNPs [50]. Plain DNA‐LNPs exhibited larger apparent intracellular sizes and dynamics consistent with near‐Brownian diffusion, albeit characterized by low diffusion coefficients, indicative of slow particle motion. In contrast, PEGylated DNA‐LNPs appeared smaller within cells and displayed faster dynamics, but with subdiffusive, spatially confined motion. Collectively, these observations suggest that PEGylation promotes the formation of smaller, more mobile intracellular DNA‐LNP populations that remain confined within specific cellular compartments, whereas non‐PEGylated systems tend to form larger assemblies with slower, less restricted intracellular transport. These observations highlight the central role of PEGylation in shaping the intracellular trafficking of DNA‐LNPs.

More recently, Renzi et al. extended this approach to investigate how protein corona formation modifies DNA‐LNP trafficking and intracellular processing [42]. It was found that coronated DNA‐LNPs can explore larger intracellular regions, consistent with a reduction in spatial confinement. Notably, this enhanced long‐range mobility of coronated particles correlates with decreased sequestration within lysosomal compartments, in agreement with confocal microscopy observations. Together, these findings highlight how subtle changes in DNA‐LNP surface identity, such as protein corona acquisition, can profoundly alter intracellular transport regimes and influence the balance between confinement and productive trafficking.

Collectively, these observations identify trafficking dynamics as a critical, yet often underappreciated, determinant of delivery efficiency.

### 3.3 Endosomal Escape Mechanisms

The third critical step is the release of the genetic payload from the endosome. This process has emerged as the most intensively studied and arguably the most critical, since nucleic acids entrapped within endosomes are otherwise degraded or recycled. Endosomal escape allows the cargo to evade the acidic, degradative environment of the endosome and reach the cytosol, from where it can be transported to the nucleus for transcription and gene expression. While recent insights are largely based on mRNA‐LNP systems [65], the concept of endosomal escape and its underlying mechanisms had already been investigated in lipoplex research in the early 2000 s, finally leading to the identification of multicomponent lipoplexes as optimal candidates for efficient gene delivery [66].

Among the proposed mechanisms facilitating endosomal escape, the first is the proton sponge effect, originally introduced by Behr [67], whereby buffering of endosomal acidification leads to osmotic swelling and rupture of the vesicle. Cationic lipids or polymers, particularly those containing protonatable amine groups, act as “proton sponges,” buffering the pH by binding incoming protons. This is accompanied by the entry of counterions and water, leading to osmotic swelling and eventual endosomal membrane disruption, releasing the nucleic acid payload into the cytosol. However, a study by Hoekstra and colleagues provided direct live‐cell imaging evidence showing that this mechanism is primarily relevant to polyplexes, whereas lipid‐mediated nucleic acid release occurs instead through lipid mixing‐induced membrane destabilization and the formation of transient pores within the endosomal membrane [68]. The second hypothesized mechanism of endosomal escape involves lipid phase transitions, in which ionizable or helper lipids adopt non‐lamellar, fusogenic structures that destabilize the endosomal membrane, allowing the release of the genetic payload into the cytosol. This phase transition can be facilitated, however, by mixing with anionic membrane lipids. Szoka and coworkers suggested that anionic membrane lipids laterally diffuse into the complex and form charge‐neutralized ion pairs with the cationic lipids [69]. This interaction weakens the binding between cationic lipids and the oligonucleotides, facilitating their displacement and eventual release into the cytoplasm. Some of us further demonstrated that the intermolecular rearrangement among cationic lipids during lipoplex formation is an entropy‐driven process governed by lipid mixing entropy [70, 71], which promotes lipid reorganization and homogeneous distribution within the membrane plane. The same entropy of lipid mixing is likely to regulate the interactions between cationic lipids and anionic membrane lipids in the endosome [59]. When such lipid‐lipid interactions take place, additional factors such as lipid shape [72] and the interfacial curvature associated with the mesomorphic structures that emerge upon mixing anionic and cationic lipids [73] are thought to contribute to the formation of membrane defects, ultimately leading to disintegration of the bilayer and release of the genetic payload. In particular, two distinct regimes were identified [74, 75]: a first regime in which anionic membrane lipids form ion pairs with cationic lipids, thereby weakening DNA‐lipid interactions while the DNA remains confined within the lipoplex and a second regime, characterized by a phase transition or by the disintegration of the original lipoplex structure, during which DNA begins to be released into the cytoplasm. Importantly, in this second regime, the shape complementarity between cationic lipids and anionic membrane lipids, together with the enrichment in specific lipid species within the lipoplex [76, 77] have been shown to facilitate lipid phase transitions or accelerate membrane disruption, contributing to what has been identified as the overall *fusogenicity* of the lipid particle. This mechanistic view is fully consistent with the live‐cell imaging studies by Hoekstra and colleagues, already discussed above [68]. Further insights from lipoplex research showed that cholesterol enhances membrane fusion and intracellular trafficking by stabilizing bilayer structures and promoting endosomal interactions [58, 77]. Consequently, incorporating cholesterol or its natural analogues is now a well‐supported design strategy for DNA‐LNPs [78].

Unlike lipoplexes, comparable knowledge for the mechanisms that govern the endosomal escape of DNA‐LNPs remains limited. While some principles may in part overlap, it would be premature to directly extrapolate conclusions from lipoplex studies to DNA‐LNPs. We therefore provide an overview of the currently available evidence on DNA‐LNP interactions with the cellular machinery, summarizing the main findings, experimental approaches, and mechanistic insights reported so far. Within this limited framework, a small number of studies have examined the contribution of ionizable lipids to endosomal escape in DNA‐LNPs. Algarni et al. used pH‐responsive ionizable lipids to enable efficient endosomal escape of DNA and subsequent gene expression [79]. Using plasmid DNA as cargo, the authors demonstrate that the choice of ionizable lipid critically influences both organ selectivity and overall transfection efficiency, with DLin‐KC2‐DMA outperforming the clinically validated DLin‐MC3‐DMA. Importantly, the findings indicate that organ‐specific gene expression is governed less by biodistribution than by lipid structure and endosomal escape properties. These results underline the need to develop DNA‐LNP formulations based on mechanisms tailored to DNA cargo, rather than assuming they behave like RNA‐based systems.

A recent study by Lotter et al. provided a comprehensive mechanistic analysis of how ionizable lipids within LNPs facilitate endosomal escape [80]. By combining confocal microscopy, fluorescence resonance energy transfer (FRET)‐based lipid mixing assays, which detect lipid rearrangements by monitoring energy transfer between closely spaced fluorescent probes and pH‐sensitive fluorescent probes, the authors suggested that protonation of ionizable lipids at acidic endosomal pH triggers the proton sponge effect. Rather than directly promoting non‐lamellar phase transitions or membrane fusion, their findings highlight the role of ionizable lipids in buffering endosomal protons and inducing membrane destabilization through increased osmotic pressure. This mechanism contributes to enhanced cytosolic release of the encapsulated payload, even though the precise morphological changes at the membrane interface were not explicitly resolved. The findings of this study do not appear to be fully consistent with the results reported by Hoekstra et al. for lipoplexes. A more precise explanation is required to determine whether this discrepancy arises from the specific role of ionizable lipids or from other, yet unidentified, mechanisms.

### 3.4 Immunological Sensing and Endosomal Fate

Recent observations suggest that lipid composition within DNA‐loaded LNPs may be asymmetrical, with distinct species localized preferentially to either the outer surface or inner aqueous core [81]. This organization could influence engagement with endosomal components and toll‐like receptors (TLRs), affecting immunogenicity and downstream signaling in a cargo‐specific manner. For instance, Tursi et al. systematically investigated how variations in the N/P ratio within DNA‐loaded LNPs influence both their biophysical properties and immunostimulatory potential [82]. Their findings revealed that increasing the N/P ratio led to improved particle homogeneity and a more favourable zeta potential, correlating with enhanced germinal center B cell responses and CD8^+^ T cell activation. Notably, DNA‐LNPs triggered a potent innate immune response via the cGAS–STING pathway, independently of TLR9 sensing, promoting a pro‐inflammatory transcriptional profile in multiple myeloid and dendritic cell subsets. This platform‐specific immune sensing not only differentiated DNA‐LNPs from mRNA‐LNPs in terms of innate recognition and endosomal escape but also translated into superior T cell memory formation and long‐lasting antigen‐specific immunity in both murine and rabbit models. These routes appear to be influenced by both particle size and surface chemistry, parameters that differ significantly between RNA‐ and DNA‐loaded formulations. A recent study by Cavegn et al. provided a quantitative and time‐resolved analysis of these dynamics by employing live‐cell imaging and EGFP‐reporter cell lines to track DNA‐loaded LNPs across endosomal escape, recycling, and lysosomal degradation [83]. Their findings revealed that LNPs achieved approximately 25% endosomal escape, with most particles (∼60%) undergoing lysosomal degradation and a smaller fraction (∼7%) being recycled. Notably, the use of endocytic recycling inhibitors such as SecinH3 further enhanced escape efficiency, highlighting how endosomal trafficking routes can be modulated pharmacologically to improve DNA delivery outcomes.

### 3.5 Nuclear Entry

The nuclear envelope represents the final and most critical barrier for plasmid DNA delivered by LNPs, particularly in non‐dividing cells where passive entry during mitosis is not possible. In addition to indirect assays, such as reporter gene expression (e.g., luciferase or GFP), which confirm transcriptional activation only after nuclear import, direct methods are necessary to mechanistically dissect DNA localization. These include fluorescent labeling of plasmid DNA combined with confocal or super‐resolution microscopy, subcellular fractionation followed by quantitative PCR (qPCR) to distinguish nuclear from cytoplasmic DNA. In the study by Kulkarni et al., LNPs containing the ionizable lipid DLin‐KC2‐DMA achieved markedly higher levels of gene expression compared to analogous formulations containing DLin‐MC3‐DMA, despite only modest differences in cellular uptake. This observation indicates that the enhanced transcriptional output arises downstream of internalization, consistent with more efficient cytosolic release of plasmid DNA rather than direct facilitation of nuclear import. Importantly, the authors emphasize that ionizable amino‐lipids are not expected to actively mediate nuclear translocation of plasmid DNA, particularly in non‐dividing cells where the nuclear envelope remains intact. Instead, their contribution lies in maximizing the number of intact plasmids released into the cytosol, thereby increasing the likelihood that a fraction of DNA molecules can access the nucleus during transient nuclear envelope remodeling associated with mitosis. In this context, optimization of lipid pKa and N/P ratio, such as the use of DLin‐KC2‐DMA at N/P ≈ 6, resulted in smaller, more compact LNPs and improved endosomal disruption, indirectly enhancing nuclear availability of plasmid DNA without invoking specific nuclear targeting mechanisms [35].

To determine whether DNA accesses the nucleus via active transport mechanisms, mutant reporter constructs lacking nuclear localization sequences (NLS) can be compared to wild‐type controls. The mechanism by which plasmid DNA delivered by LNPs enters the nucleus—particularly in non‐dividing cells—can also be studied by adopting the experimental strategy developed by Ferri et al. for lipoplexes [84]. Their multi‐channel 3D confocal imaging approach, which combines Hoechst staining of the nucleus with FM4‐64 spectral imaging of the nuclear envelope, enables precise localization of DNA relative to nuclear structures. Using fluorescently labeled DNA, they observed its accumulation at tubular invaginations of the nuclear envelope, suggesting these regions as potential entry sites mediated by membrane remodeling.

In sum, while some mechanisms of cellular entry and endosomal escape are shared between RNA‐ and DNA‐loaded LNPs, DNA systems face distinct barriers that necessitate targeted optimization. Lipid identity, charge ratio, and structural design must be co‐optimized not only to enhance uptake and cytoplasmic release, but also to ensure that DNA successfully reaches the nucleus to exert its intended effect. On the other hand, the high level of mechanistic detail that has been achieved for lipoplexes [81] and that paved the way for the development of RNA‐LNPs and, ultimately, COVID‐19 vaccines, is still lacking for DNA‐LNPs. It cannot be assumed that DNA‐LNPs behave in the same way as lipoplexes, given their structural and physicochemical differences. Unlike lipoplexes, which rely on permanently charged cationic lipids to condense DNA, LNPs are typically based on ionizable lipids that are only protonated at acidic pH. This fundamental distinction likely affects the strength and reversibility of DNA‐lipid interactions, with obvious implications for endosomal escape and cytoplasmic release.

## 4 Bio‐Nano Interface of DNA‐Loaded LNPs: Role of Biomolecular Corona in Delivery Efficacy and Immunomodulation

When nanomaterials enter biological environments such as blood, they are rapidly enveloped by a dynamic layer of biomolecules, predominantly proteins, known as the biomolecular corona, as originally termed by Dawson and coworkers [85]. This corona redefines the synthetic identity of the pristine material into a biological identity, profoundly influencing its colloidal stability, opsonization, immune recognition, biodistribution, and cellular uptake [86]. As in the previous sections, we will first present the results obtained on lipid‐based systems that preceded and paved the way for LNPs in the field of gene delivery. In 2010, the first results were obtained by our group at the NanoDelivery Lab, Sapienza University of Rome, which was the first to extend Dawson's pioneering concept of the protein corona—originally developed for inorganic NPs such as polystyrene—to lipid‐based systems including lipoplexes [87] and liposomes [88]. These studies demonstrated that protein adsorption fundamentally redefines the biological identity of lipid carriers, thereby laying the groundwork for subsequent research on DNA‐ and RNA‐loaded LNPs. They also introduced the notion that protein adsorption is not a passive process but a key determinant of the biological fate of lipid‐based nanocarriers. This early insight is particularly remarkable in light of recent evidence showing that the corona of LNPs can serve as an endogenous functionalization, actively guiding their biodistribution in vivo [42].

A paradigmatic example of how the protein corona can endow lipid‐based carriers with targeting properties was reported more than a decade ago with DOTAP/DNA lipoplexes [89]. When incubated with human plasma, these nanostructures were found to spontaneously recruit large amounts of vitronectin, which accounted for over 30% of the corona composition. Vitronectin, in turn, acted as a functional ligand for cancer cells overexpressing the ανβ3 integrin receptor, hereby promoting selective cellular uptake and shifting the internalization pathway from nonspecific endocytosis to receptor‐mediated entry. These findings paved the way for introducing the concept that the protein shell acquired in vivo is not merely a passive coating but can function as an endogenous targeting element, foreshadowing the idea that drug delivery systems could exploit the corona itself to achieve selective tropism [90].

Several years later, the concept that the protein corona could be exploited to modulate biodistribution was further developed with the introduction of the so‐called selective organ targeting (SORT) NPs for mRNA delivery [91]. These systems were specifically engineered to alter their in vivo fate by tuning corona composition, thereby redirecting LNPs to distinct tissues. Of particular relevance, the cationic version of SORT NPs was formulated primarily with DOTAP, clearly echoing earlier observations [89] and reinforcing the notion that surface charge and protein adsorption are central determinants of biological identity and targeting.

More recently, this concept has been extended to tumor targeting. A study by Vaidya and colleagues [92] confirmed that LNPs engineered to recruit vitronectin from plasma can exploit the same mechanism originally reported for DOTAP/DNA lipoplexes [89]. Vaidya et al. showed that DOTAP‐containing LNPs enriched in vitronectin achieved efficient endogenous targeting of kidney tumors overexpressing αvβ3 integrin, enhancing mRNA delivery both in vitro and in patient‐derived orthotopic xenografts. These findings establish a direct line of continuity between the pioneering evidence of corona‐mediated targeting of cancer cells by vitronectin‐decorated lipoplexes and the most recent demonstrations that LNPs can harness the same vitronectin‐integrin interaction to achieve tumor‐selective biodistribution.

While these studies have become highly popular and widely accepted in the scientific literature, it is important to recognize some inherent limitations. A first challenge lies in the attempt to correlate in vivo biodistribution with ex vivo corona composition, typically determined after static incubation of NPs with biological fluids in cuvettes, followed by mass spectrometry. Several reports have shown that static and dynamic incubations, the latter mimicking blood flow [93], can yield significantly different corona profiles. Moreover, in a translational perspective, it must be remembered that the corona formed in murine plasma [94] or even recovered from animals [95] is again different. Correlating human corona profiles with murine biodistribution is a common strategy that may, however, introduce artifacts. Thus, while some general principles may hold true, the only certainty is where particles ultimately accumulate in vivo as a function of their formulation. The mechanistic interpretation that a given protein mediates targeting remains speculative, as we still lack definitive knowledge of the actual in vivo corona composition, the 3D organization of proteins around the NP, and the true accessibility of epitopes to engage cellular receptors [96]. Our intent here is not to discourage the field, but rather to emphasize that real progress will depend on addressing these methodological challenges [97]. Recognizing them in depth, even if they cannot yet be fully overcome, should provide essential guidance for the future directions of research.

As with other physicochemical properties of LNPs, the biomolecular corona has been thoroughly investigated in RNA‐loaded LNPs, while significantly less attention has been paid to corona formation in DNA‐loaded LNPs. This gap is not trivial, as the nature of the nucleic acid cargo itself may indirectly affect protein adsorption on the NP surface. In the previous sections, we have clarified how the larger size and higher rigidity of plasmid DNA compared to smaller RNA species can alter particle architecture, surface charge distribution, and overall stability. These effects are likely to influence the lipid arrangement at the surface of the LNP, for example, by redistributing lipid species within the outer leaflet and modifying their lateral organization in the membrane plane. Similar questions have been extensively investigated in the context of lipoplexes, where detailed structural and biophysical studies clarified how lipid rearrangement governs particle behavior. Such reorganizations, together with peculiar physical‐chemical properties of DNA‐LNPs, could reshape the physicochemical interface that governs protein adsorption and, in turn, substantially affect the composition and organization of the protein corona, potentially leading to fingerprints distinct from those of RNA‐LNPs.

**FIGURE 3:**
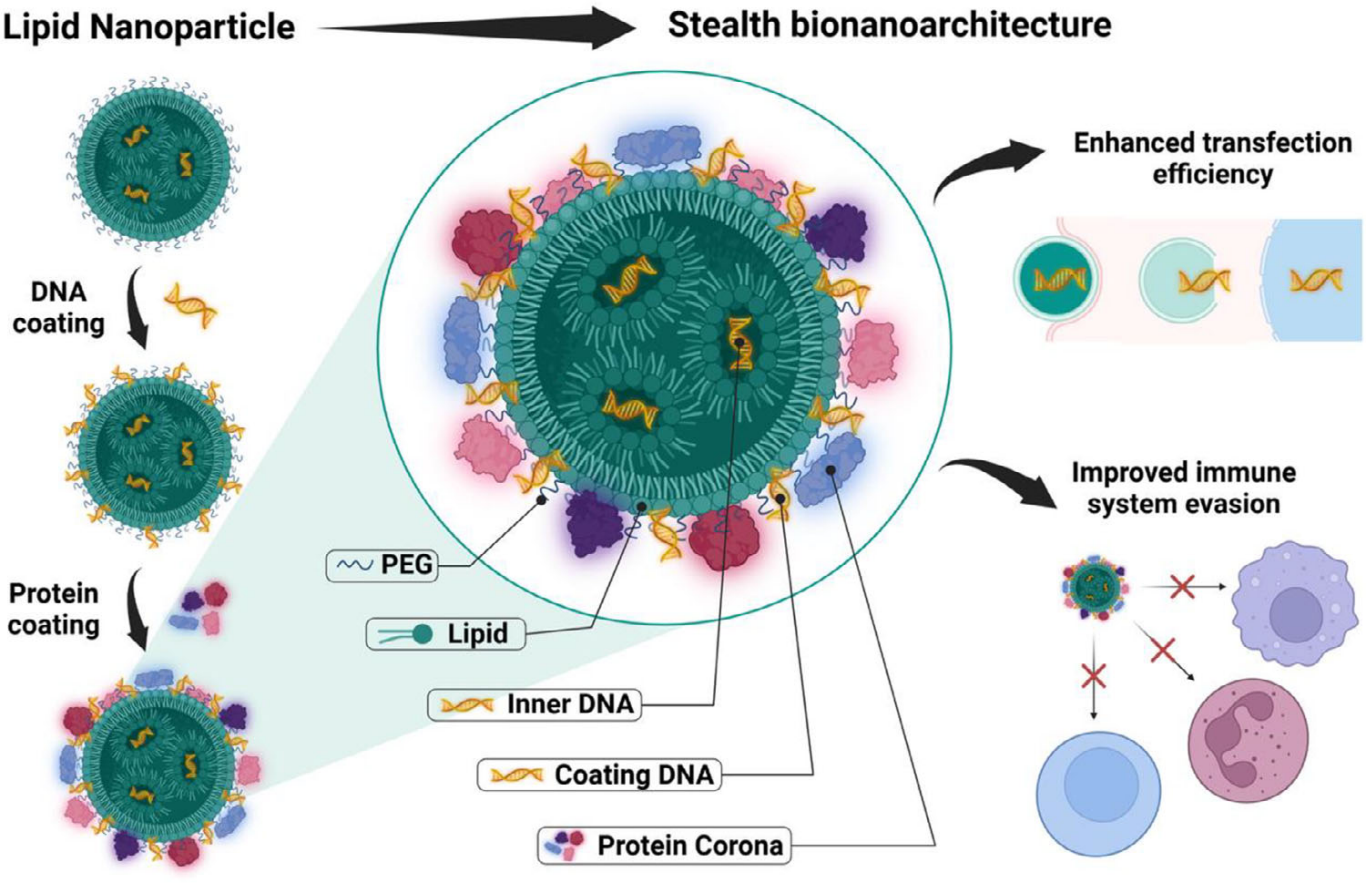
From DNA‐LNPs to stealth bio‐nanoarchitectures. Schematic illustration of the transformation of DNA‐loaded lipid nanoparticles (DNA‐LNPs) into stealth bio‐nanoarchitectures upon interaction with biological fluids. Following exposure to plasma, DNA‐LNPs undergo sequential surface remodeling driven by DNA coating and subsequent protein adsorption, leading to the formation of a biomolecular corona. This corona consists of a dynamic and composition‐dependent layer of plasma proteins that defines the nanoparticle's biological identity and governs its recognition, trafficking, and clearance in vivo. The physicochemical properties of the LNP core—including lipid composition, PEG content, surface charge, and lipid‐to‐DNA (N/P) ratio—critically regulate corona formation. Highly cationic surfaces and low N/P ratios favor the adsorption of adhesive proteins such as vitronectin, which can engage integrin receptors (e.g., αvβ3) through RGD motifs, thereby enhancing cellular binding and uptake. In contrast, DNA surface coating and near‐neutral surface charge promote the recruitment of fewer opsonins, such as immunoglobulins and complement factors, resulting in reduced immune recognition and improved immune evasion. The interplay between corona composition, cellular affinity, and immune invisibility ultimately determines biodistribution, organ targeting, and transfection efficiency, highlighting protein corona engineering as a key strategy for the rational design of next‐generation DNA‐LNP delivery systems. (Created with BioRender.com).

Another possibility to consider is that plasmid DNA may not always remain fully encapsulated within the NP core. While RNA cargos are generally compact and confined to the interior of LNPs, resulting in a predominantly lipidic surface where protein adsorption is dictated mainly by the lipid composition, plasmid DNA, particularly at low lipid‐to‐DNA molar ratios, can partially localize at the particle surface. In such cases, the surface‐exposed DNA becomes an integral component of the interfacial landscape, directly modulating charge distribution and potentially leading to the formation of more anionic particles. This scenario could dramatically influence the corona, producing protein adsorption profiles that diverge from those observed in RNA‐LNPs. A recent study exploited the surface localization of DNA to demonstrate that DNA‐decorated LNPs preferentially adsorb a low‐opsonin biomolecular corona, thereby reducing uptake by immune cells and outperforming conventional PEGylation strategies in terms of immune evasion [42]. This effect appears to be critically dependent on the net negative charge conferred by the nucleic acid coating, in contrast to LNPs with predominantly cationic lipid surfaces, which tend to recruit opsonins (e.g. immnunoglobulins and complement proteins) and are more readily internalized by phagocytic cells. Notably, this principle had already emerged in earlier work on liposome coronas, where distinct protein adsorption profiles were linked to cationic, anionic, or neutral surface charges, ultimately associating surface charge with differential biological identity and fate [98]. As a result, DNA‐decorated nanoconstructs have shown enhanced transfection efficiency and safety in controlled laboratory studies, together with improved immune evasion in vivo. These findings provide important insights for the rational design of bionanoarchitectures for large DNA delivery, opening promising avenues toward transformative gene therapies (Figure 3).

A similar approach has been proposed by Nabar et al., who developed a platform based on the electrostatic adsorption of biocompatible polyanions onto DNA‐ or RNA‐loaded LNPs [99]. By layering LNPs with different classes of polyanions, such as hyaluronate (HA), poly‐L‐glutamate (PLE), and poly‐L‐aspartate (PLD), the authors achieved tunable modifications of the NPs’ surface charge, pKa, and protein adsorption profile. This modular coating strategy significantly reduced opsonin binding and altered the intracellular trafficking and transfection kinetics in a cell‐type‐specific manner. Notably, HA‐layered LNPs enhanced plasmid DNA transfection in epithelial and immune cells, while PLE‐coated systems improved in vivo gene expression in liver and spleen tissues without increasing toxicity. In line with Renzi et al., the study of Nabar et al. demonstrates that negative electrostatically adsorbed biomolecules can act as stealth and targeting agents at once, enabling the rational design of LNP surfaces to improve delivery specificity and reduce off‐target uptake.

Insights on how biomolecular coronas form and evolve on DNA‐loaded LNP were provided by Wang et al. [100]. In their study, the authors investigated how different culture conditions, specifically the presence or absence of serum and amino acids, modulate the composition of the corona and, consequently, the intracellular fate of LNPs carrying FITC‐labeled plasmid DNA. Using confocal imaging and live‐cell assays in HeLa cells, they found that LNPs incubated in complete medium (containing serum and nutrients) predominantly localized in perinuclear compartments after 4 h, a behavior mediated by cytoskeletal elements such as F‐actin and coronin‐1A. This suggests that serum‐derived coronas actively promote actin‐dependent intracellular transport, favoring nuclear‐adjacent accumulation that may support more efficient gene delivery. Conversely, under serum‐free or amino acid‐deficient conditions, the LNPs failed to accumulate perinuclearly and instead exhibited diffuse cytosolic distribution, indicating a disruption in the typical trafficking pathway. Taken together, the results reviewed in this section reveal that DNA‐LNPs acquire a biomolecular corona with properties and biological consequences that diverge significantly from those of RNA‐LNPs. Larger size altered surface charge distribution, partial surface exposure of plasmid DNA, and increased structural heterogeneity collectively reshape the identity of DNA‐LNPs at the bio‐nano interface. These differences affect opsonization patterns, immune recognition, intracellular routing, and ultimately the efficiency and safety of gene transfer. Table 1 summarizes these distinctions across corona composition, immune engagement, and downstream cellular behavior, making explicit that DNA‐LNPs cannot rely on the same biological assumptions that have guided RNA‐LNP development.

## 5 Conclusions

While RNA‐based LNPs have already reached clinical maturity, supported by well‐established formulation principles, comparable knowledge is still lacking for DNA‐loaded systems. This review emphasizes the limitations of directly applying RNA design paradigms to DNA cargos and calls for a cargo‐specific framework for developing effective nanocarriers. On the other hand, it is the responsibility of researchers with long‐standing experience in the field to guide the next generation by highlighting what has already been achieved in related systems, such as lipoplexes, and providing a clear framework for designing and interpreting future investigations. Among these, special attention should be given to the protein corona, which has emerged as a crucial determinant of LNP behavior and performance in vivo.

In conclusion, the field of DNA‐loaded LNPs is rapidly evolving from a marginal offshoot of RNA research into a domain with its own distinct intracellular processing [83]. By embracing the complexity of the DNA cargo and its interface with lipids, cells, and serum proteins, researchers are poised to unlock the full therapeutic potential of non‐viral gene delivery. The coming years will likely see an acceleration in the development of personalized, programmable nanocarriers capable of delivering DNA with precision, safety, and functional durability. Such advances could unlock transformative opportunities in gene therapy for monogenic disorders, in the engineering of patient‐specific cell therapies, in regenerative medicine approaches relying on controlled expression of therapeutic genes, and in the development of novel DNA vaccines. By refining our understanding of DNA‐LNP biology and delivery mechanisms, these technologies may ultimately broaden the therapeutic reach of nucleic acid nanomedicine well beyond what has been achieved with RNA‐based systems.

## Funding

The authors have nothing to report.

## Conflicts of Interest

The authors declare no conflict of interest.

## Data Availability

The authors have nothing to report.
